# Sex-specific association patterns in bonobos and chimpanzees reflect species differences in cooperation

**DOI:** 10.1098/rsos.161081

**Published:** 2017-05-03

**Authors:** Martin Surbeck, Cédric Girard-Buttoz, Christophe Boesch, Catherine Crockford, Barbara Fruth, Gottfried Hohmann, Kevin E. Langergraber, Klaus Zuberbühler, Roman M. Wittig, Roger Mundry

**Affiliations:** 1 Department of Primatology, Max Planck Institute for Evolutionary Anthropology, Deutscher Platz 6, 04103 Leipzig, Germany; 2 Liverpool John Moores University, Faculty of Science, Natural Sciences and Psychology, Liverpool, UK; 3 Centre for Research and Conservation, Royal Zoological Society of Antwerp, Antwerp, Belgium; 4 School of Human Evolution & Social Change, Arizona State University, Tempe, AZ, USA; 5 Institute for Human Origins, Arizona State University, Tempe, AZ, USA; 6 Budongo Conservation Field Station, Masindi, Uganda; 7 Cognitive Science Centre, University of Neuchâtel, Neuenburg, Switzerland; 8 Taï Chimpanzee Project, Centre Suisse de Recherches Scientifiques, BP 1303 Abidjan 01, Côte d'Ivoire; 9 Max Planck Institute for Evolutionary Anthropology, Deutscher Platz 6, 04103 Leipzig, Germany

**Keywords:** sociality, sexual segregation, competition, kinship, *Pan troglodytes*, *Pan paniscus*

## Abstract

In several group-living species, individuals' social preferences are thought to be influenced by cooperation. For some societies with fission–fusion dynamics, sex-specific association patterns reflect sex differences in cooperation in within- and between-group contexts. In our study, we investigated this hypothesis further by comparing sex-specific association patterns in two closely related species, chimpanzees and bonobos, which differ in the level of between-group competition and in the degree to which sex and kinship influence dyadic cooperation. Here, we used long-term party composition data collected on five chimpanzee and two bonobo communities and assessed, for each individual of 10 years and older, the sex of its top associate and of all conspecifics with whom it associated more frequently than expected by chance. We found clear species differences in association patterns. While in all chimpanzee communities males and females associated more with same-sex partners, in bonobos males and females tended to associate preferentially with females, but the female association preference for other females is lower than in chimpanzees. Our results also show that, for bonobos (but not for chimpanzees), association patterns were predominantly driven by mother–offspring relationships. These species differences in association patterns reflect the high levels of male–male cooperation in chimpanzees and of mother–son cooperation in bonobos. Finally, female chimpanzees showed intense association with a few other females, and male chimpanzees showed more uniform association across males. In bonobos, the most differentiated associations were from males towards females. Chimpanzee male association patterns mirror fundamental human male social traits and, as in humans, may have evolved as a response to strong between-group competition. The lack of such a pattern in a closely related species with a lower degree of between-group competition further supports this notion.

## 1. Introduction

Animals living in socially cohesive societies experience trade-offs associated with group living. While group living provides benefits such as enhanced defence against predators, access to mating partners and food defence [1–3], it also entails costs such as increased mating and feeding competition, higher risk of disease transmission and of infanticide [3–6]. Group living can evolve only when the benefits outweigh the costs of cohesive spatial association with conspecifics [3]. Animals adopt behavioural strategies to optimize the associated cost/benefit trade-offs. In particular, a growing body of evidence suggests that associating and/or affiliating preferentially with certain conspecifics over others impacts individual fitness [7–10]. An individual's choice of association partner is therefore crucial, especially in species with a high degree of fission–fusion dynamics [11]. In such species, the social group regularly splits into subgroups (hereafter parties) which temporally vary in size and composition, often as a response to spatio-temporal changes in food availability and predation pressure [12–19]. Therefore, the opportunity to affiliate and cooperate with group members is limited to the conspecifics present in those parties. While association patterns within parties are not a direct measure of the cooperation between specific individuals, these patterns reflect the opportunity for associates to cooperate with each other (e.g. [20,21]). Cooperation such as alliance formation [22–24] might enable individuals to outcompete rivals during within-group competition for fitness-limiting resources, which vary between the sexes (access to food for females and access to mating partners for males [25–27]).

Selectivity in association partners has been found in several mammalian species with a high degree of fission–fusion dynamics (e.g. spotted hyenas, *Crocuta crocuta* [20], giraffes, *Giraffa camelopardalis* [28] and Bechstein's bat, *Myotis bechsteinii* [29]). In these species, association patterns are often strongly kin-biased (e.g. Bechstein's bat [29], elephants, *Loxodonta africana* [30] and bottlenose dolphins, *Tursiops aduncus* [31]) since, in addition to direct benefits, individuals derive indirect fitness benefits by associating and cooperating with kin [32]. Yet, when the availability of kin partners is limited and/or when kin do not have the required skills or attributes to achieve a given goal, selectively associating with non-kin might also provide benefits especially when familiarity and frequent proximity allows for repeated cooperation within a dyad, leading to shared benefits (e.g. in bottlenose dolphins [33] and chimpanzees, *Pan troglodytes* [34–36]). Such selective associations are likely to be particularly adaptive in species where dyadic forms of cooperation within groups are beneficial. While within-group competition plays a role in shaping association patterns, between-group competition can influence within-group social dynamics [37] and constrain association patterns. In species with frequent aggressive inter-group encounters (i.e. high between-group competition), the number of individuals involved in those encounters can be a key parameter in determining the success of territory defence [38]. Given the need to cooperate with many individuals in such a context, it is important to maintain close association with many possible cooperation partners [39]. Therefore, it is more beneficial to associate less selectively with a larger number of individuals than to associate more selectively with a smaller number of individuals. However, under such conditions, association preferences are also expected to be strongest among the sex that is most involved in territory defence. For example, in species where territory defence is principally undertaken by males (e.g. chimpanzees [40] and spider monkeys, *Ateles* spp*.* [41,42]), outgroup pressure can push males to associate more with other males leading to sexually segregated association patterns [21,43].

Male chimpanzees jointly defend their territory, but they also hunt cooperatively and support each other during within-group conflict, whereas female chimpanzees do so less regularly ([44], but see also [45]). Based on those differences, some authors argued that the tendency for chimpanzee male–male dyads to associate more (i.e. spend more time in the same subgroup or party) than female–female dyads might be directly linked to the difference in the intra-sexual level of cooperation among male and among female chimpanzees [21]. Gilby & Wrangham's [21] argument that association patterns are driven by cooperative needs can be extended to predict differences in sex-specific patterns of association between chimpanzees and a closely related species [46], the bonobo (*Pan paniscus*). Despite their similar social and genetic structure (multi-male multi-female communities with high fission–fusion dynamics and males being mainly philopatric [47–50]), bonobos and chimpanzees differ in the ways in which sex and close maternal kinship influence dyadic cooperation during within-group competition. While most of the alliances are formed between males (both related and unrelated) in chimpanzees [34,51,52], female–female coalitions are the most frequent form of alliances found in bonobos, and males rarely cooperate with each other [53,54]. Instead, male bonobos are mainly supported by their mother [48,55–57], a pattern occasionally observed in chimpanzees [23]. In addition, chimpanzees and bonobos differ in their level of between-group competition. While chimpanzees are highly territorial, with hostile inter-group encounters that can be lethal [58,59], bonobos’ territoriality and hostility is less pronounced and inter-group encounters have never been reported to be lethal [59–61]. In our study, we took advantage of these species differences to extend the test of Gilby and Wrangham's hypothesis that the potential for cooperation drives association patterns [21]. This was previously tested on association patterns of a single community of chimpanzees (their study). Here, we include two closely related species and multiple communities per species.

Several studies have been conducted on sex-specific association patterns in bonobos and chimpanzees, highlighting possible species differences in association dynamics between the two *Pan* species. Studies of different chimpanzee populations show that males consistently associate more with one another than females do with females and males do with females [21,23,62–65]. The picture is less clear when comparing male–female and female–female association strength. While in Gombe and in one study in Kanyawara male–female association values were higher than female–female values [49,64], the opposite was found in Mahale and in another study in Kanyawara [66,67], and no significant differences were found between male–female and female–female associations in Kalinzu [65] and in Taï [23,68]. These discrepancies could be related to true differences in the levels of female–female cooperation or benefits of associations between mothers across the different communities. It could be also the trade-offs females face when associating with males related to variation in the degree of feeding competition and males' propensity to aggress and/or support females [69,70]. However, they might also be partially or even entirely driven by methodological differences between studies (see below).

Association patterns have been much less studied in bonobos, but available data suggest some clear differences in sex-specific associations between the two species. In contrast with the general chimpanzee pattern, female–female associations were the most frequent associations in bonobos in Lomako followed by male–female associations, and then male–male associations [60,71]. However, in another study on the bonobo population of Wamba, using different methods, no significant differences were found in the degree of association between the different sex combinations [65].

The studies conducted so far on sex-specific association patterns in bonobos and chimpanzees provide a basis for our understanding of social relationships and social structure in these two species. Yet the picture is incomplete and partly inconsistent, possibly also due to a lack of standardization in the methodological approaches. For instance, randomization methods to assess whether the association patterns observed differ from random association have been applied only in some chimpanzee studies and in only one bonobo study. Furthermore, when randomization procedures were applied, the party composition was randomized without taking into account each individual's level of gregariousness (i.e. by only reshuffling individuals within parties of the same size). Thus, the indices used may reflect whether two individuals are more or less likely to associate than by chance given the gregariousness of the study group but not of each individual. Gregariousness, however, can vary between species and between populations of the same species due to several factors unrelated to association preferences (e.g. predation pressure [62], food availability [72]). Therefore, it is crucial to control for gregariousness since this parameter can significantly alter the results [73]. More specifically, controlling for gregariousness allows for addressing questions regarding individuals' preferences to associate with specific group members over others beyond the mere effect of their general sociality. To overcome these issues, we used long-term datasets from five chimpanzee and two bonobo communities from five different field sites. After standardizing party composition data across datasets, we applied randomization methods controlling for differences in individual gregariousness and observation time. We assessed the difference between bonobos and chimpanzees in sex-specific association patterns within a single analytical framework to test whether their association patterns were primarily driven by cooperative needs. For each individual, we determined the sex of its top associate and of all conspecifics with whom it associated more than by chance (hereafter ‘significant associates’). It is important to note here that we did not test for differences in gregariousness itself. While top associates provide information about preference of individuals to associate with a given group member of a given sex over all others, information about significant associates allows for addressing questions pertaining to the general tendency for male and female bonobos and chimpanzees to associate more with other males or females. In our analysis we also assessed how skewed were the dyadic associations within each sex combination, i.e. for each individual we assessed whether it associated more uniformly or more selectively with all other individuals of a given sex.

Based on the general hypothesis that association preferences are driven by cooperative needs in chimpanzees and bonobos and given the sex-specific structure of cooperation in both species described above, we predict the following:

In chimpanzees known to exhibit strong between-group competition and high levels of male–male cooperation, we expected that males have principally other males as top and significant associates. Given the potential benefits of socialization of offspring with a same-aged peer [74,75], we expected females to have other females as top and significant associates. Furthermore, we expected a limited impact of close maternal kinship (mother–offspring and maternal siblings) on those association patterns among females because of female dispersal [76], and among males because large inter-birth intervals reduce the availability of suitable closely related cooperation partners [51].

In bonobos, with low aggression rates during between-group encounters, low levels of male cooperation, strong mother support towards their sons and relatively high levels of female–female cooperation, we expect that males will not exhibit a bias towards either sex as top and/or significant associates when close maternal kinship is controlled for but will exhibit a bias towards females as top and/or significant associates (i.e. their mothers) when this factor is not controlled for. Finally, we expect females to have mostly other females as top and significant associates.

## 2. Material and methods

### 2.1. Party composition data

We compiled datasets originating from long-term studies on five chimpanzee and two bonobo communities. For chimpanzees, these comprised three communities of western chimpanzees (Taï North, Taï South and Taï East, Taï National Park, Côte d'Ivoire) and two communities of eastern chimpanzees (Sonso community in Budongo Forest and Ngogo community in Kibale National Park, Uganda). The bonobo data came from two communities, the Bompusa community in LuiKotale and the Eyengo community in Lomako, both situated in Democratic Republic of Congo. Overall, data span over periods from 2 to 20 years per study community (see table 1 for details).

**Table 1.** Summary of the seven datasets included in the study. ‘F’ indicates females and ‘M’ indicates males.

				number of adult males			number of adult females			% of dyads which were close maternal kin (% of dyads which were mother–offspring)		
study site	study community	study species	observation period	median	min	max	median	min	max	F–F	M–F	M–M
Taï	Taï North	chimpanzee	1992–2012	2	1	6	7	2	13	3.1 (3.1)	4.1 (2.7)	1.9
Taï	Taï South	chimpanzee	2000–2012	4.5	2	8	10	6	14	0.4 (0.4)	2.0 (2.0)	0
Taï	Taï East	chimpanzee	2009–2012	5.5	5	6	11	10	12	0 (0)	0 (0)	0
Budongo	Sonso	chimpanzee	2007–2013	14	13	17	26.5	12	33	1.7 (1.7)	2.5 (1.5)	0.4
Kibale	Ngogo	chimpanzee	2003–2004	37	19	38	42.5	15	49	0.5 (0.4)	1.2 (1.0)	0.5
Lomako	Eyengo	bonobo	1990–1998	7	5	8	14	5	17	0 (0)	4.1(3.4)	10.3
LuiKotale	Bompusa	bonobo	2007–2013	7	4	9	13	12	16	0.3 (0.3)	4.8 (4.8)	3.9

For our analysis of party composition, we included only individuals 10 years of age and older since 9.5 years is the youngest age at which chimpanzees and bonobos were ever recorded to reproduce in the wild [77,78]. With the exception of Lomako, hourly party compositions (i.e. the identity of all individuals present in the party within a given hour) were extracted from the long-term data of the study communities [79]. At Lomako, the party composition was only recorded when all individuals of a given party were clearly visible to the observer, resulting in a larger time lag between consecutive party records than one hour. While differences in the party composition-recording protocol potentially result in differences in actual party sizes and individual gregariousness, we explicitly do not compare the species in these parameters but rather focus on within-community differences in dyadic association strengths.

#### 2.1.1. Maternal relatedness

For all communities, we used a combination of published (Taï [80], Budongo [81,82], Kibale [51], Lomako [83,84], LuiKotale [55,84,85]) and unpublished genetic and demographic data to identify close maternal kin (chimpanzees: 1.1% of female–female dyads, 2.0% of male–male dyads, 0.6% of male–female dyads; bonobos: 0.2% of female–female dyads, 4.5% of male–male dyads, 7.1% of male–female dyads; for more details see table 1). We focused on maternal kinship here since, at least in chimpanzees, paternal kinship may not affect social preferences [51]. As described in these previous publications, up to 44 autosomal microsatellites were used in likelihood-based CERVUS parentage analyses [86] to identify mothers. Maternal siblings were in turn identified as those individuals who shared the same mother. The rationale here is, first, most females emigrate from their natal community to join a new community at adolescence, and second, previous research in one chimpanzee community has shown that the vast majority of female dyads are not maternal siblings or mother–offspring pairs [76]. Thus, we defined any dyad among adult females that was not observed from birth to have the same mother or was not observed as a mother–daughter dyad to be unrelated. Moreover, for many male–male and male–female dyads we were unable to determine whether or not they were maternal siblings, as their mothers died before sample collection and genotyping were possible.

Owing to these methodological limitations, our analyses regarding the effects of close maternal kinship on association patterns should be viewed with caution, especially as populations of both species seem to vary mildly in their degree of female dispersal [50,85,87].

#### 2.1.2. Overview data analysis

After characterizing the strength of all dyadic associations in each community of our dataset, we fitted (i) two generalized linear mixed models (GLMMs) to test for species differences in the sex combinations of close associates, (ii) two GLMMs to test for species differences in the determinants of close associates and (iii) a linear mixed model (LMM) to assess species differences in the degree of differentiation in association between partners of different sex combinations (table 2).

**Table 2.** Structure of all the models used in the analysis.

	sex-top-associates model (Model 1a)	sex-significant-associate model (Model 1b)	what-makes-top-associates model (Model 2a)	what-makes-significant-associates model (Model 2b)	association-skew model (Model 3)
response	sex of the top	sex of the	Was individual 1 top	Was individual 1	skew index of the PAV
	associate	significant	associate of	significant associate	distribution (SK_PAV_)
		associates	individual 2 (Y/N)	of individual 2 (Y/N)	
fixed factors^a^	sex of the individual species (chimpanzee/bonobo) species × sex		sex of individual 1 sex of individual 2 species (bonobo/chimpanzee) Are individuals 1 and 2 kin? (N/Y) Were individuals 1 and 2 top/significant associate in the previous quarter? (N/Y) Sex 1 × Sex 2 × species kin × species top/significant associate in the previous quarter × species		sex of individual 1 sex of individual 2 species (bonobo/chimpanzee) Sex 1 × Sex 2 × species
random intercepts	individual ID community ID quarter		individual 1 ID individual 2 ID dyad individual 1–individual 2 community ID		individual 1 ID quarter ID community ID quarter | individual 1 ID
random slopes^b^	sex | community ID sex | quarter		kinship | individual 1 ID kinship | individual 2 ID kinship | community ID sex individual 1 | individual 2 ID sex individual 1 | community ID sex individual 2 | individual 1 ID sex individual 2 | community ID top/significant associate in the previous quarter | individual 1 ID top/significant associate in the previous quarter | individual 2 ID top/significant associate in the previous quarter | community ID		sex individual 1 | quarter sex individual 1 | community ID sex individual 2 | quarter sex individual 2 | community ID sex individual 2 | individual 1 ID Sex 1 × Sex 2 | community ID Sex 1 × Sex 2 | quarter
offset	proportion of males in the community				

^a^Sex was dummy coded with females being the reference category; species was dummy coded with bonobo being the reference category; kinship was dummy coded with non-kin being the reference; and ‘top/significant associate in the previous quarter’ was dummy coded with no being the reference category.

^b^For inclusion as random slopes we manually dummy coded and then centred (to a mean of zero) kinship, sex and ‘top/significant associate in the previous quarter’.

### 2.2. Characterizing the strength of dyadic associations

To characterize the strength of association between two individuals (i.e. dyadic association), we first calculated an observed simple ratio index (SRI_obs_, this index is similar to the ‘index of familiarity’ proposed by Nishida [47]). For each dyad, *A* and *B*, SRI_obs_ was calculated as follows:

$$\mathrm{SR}I_{\mathrm{obs}}=\frac{\mathrm{Pa}(AB)}{\mathrm{Pa}(A)+\mathrm{Pa}(B)-\mathrm{Pa}(AB)},$$

where Pa(*AB*) is the number of parties comprising both individual *A* and individual *B*, Pa(*A*) is the number of parties including individual *A* and Pa(*B*) is the number of parties including individual *B*.

We then calculated an expected SRI value (SRI_exp_) under the null hypothesis that individuals associate at random. SRI_exp_ was calculated as SRI_obs,_ but Pa(*AB*), Pa(*A*) and Pa(*B*) values were calculated based on randomized datasets (see below).

To quantify to which extent the observed association pattern of each dyad differed from random association, we subsequently calculated a pairwise affinity value (PAV) by subtracting the expected from the observed SRI (for the other procedure, e.g. calculating the ratio between observed and expected value [73]). To standardize the resulting value to show dyads that were together as little as possible to as much as possible (range −1 to 1, respectively), we derived the association score as follows:

$$\mathrm{PAV}=\frac{\left(\mathrm{SR}I_{\mathrm{obs}}-\mathrm{SR}I_{\exp}\right)}{\left(1-\mathrm{SR}I_{\exp}\right)},\;\mathrm{if}\;(\mathrm{SR}I_{\mathrm{obs}}-\mathrm{SR}I_{\exp})>0$$

and as

$$\mathrm{PAV}=\frac{\left(\mathrm{SR}I_{\mathrm{obs}}-\mathrm{SR}I_{\exp}\right)}{\mathrm{SR}I_{\exp}},\;\mathrm{if}\;(\mathrm{SR}I_{\mathrm{obs}}-\mathrm{SR}I_{\exp})<0.$$

By definition, dyads with PAVs larger than zero were observed more often in the same party than expected by chance, and dyads with a PAV smaller than zero were observed less often in the same party than expected by chance. For each individual, we defined the ‘top associate’ as the partner with whom it shared the highest PAV. If the expected value was larger than the observed value in less than 50 randomizations (out of 1000 randomizations of party compositions; see below), a dyad was considered to be significantly more often observed together than expected by chance; we refer to such pairs as ‘significant associates’. Top and significant associates are not mutually exclusive: while one of the significant associates of a given individual is the top associate, not all top associates are necessarily significant associates. For each community, except for Lomako, the datasets were split into three-month periods and the PAV, top associates and significant associates were determined separately for each three-month period (hereafter these three-month periods are referred to as ‘quarters’). In Lomako, the sample size was too small to achieve meaningful estimates of PAVs within a three-month period, so we calculated one value per year.

To account for individual differences in gregariousness, community-specific party sizes, the frequency with which individuals were observed and autocorrelation between consecutive party observations [88], we used three different randomization algorithms (‘individual’, ‘blockwise’ and ‘subset’ randomizations) and conducted all the procedures described below for each of the three sets of PAVs and number of significant associates. Details about the randomization methods are given in the supplementary information.

We subsequently used the PAVs, top associate and significant associates derived from all three randomization methods separately and compared their outcomes. We fitted a series of several GLMMs and LMMs [89] to assess whether bonobos and chimpanzees differed in the sex combinations of the top and significant association partners (male–male, male–female and female–female) and in the differentiation of the PAVs for each sex combination (indicative of selectivity). We also used mixed models to assess whether the effect of maternal kinship on association patterns differed between the two species. A summary of all models' structure is provided in table 2.

### 2.3. Species differences in the sex of top and significant associates

To test for the hypothesis that general sex-specific association preferences differ between chimpanzees and bonobos, we fitted two GLMMs, one with the sex of the top associate (Model 1a: sex-top-associate model) and one with the sex of the significant associates (Model 1b: sex-significant-associate model) of a given individual during a given quarter (except for the Lomako data where the analysis was conducted per year) as the response variable (0 = female, 1 = male) and using a binomial error distribution and logit link function [90]. For both models, we included as test predictors species and its interaction with the sex of the individual. To account for differences in the availability of both sexes during a given quarter (year in the case of Lomako), we included the logarithm of the proportion of males in the community (determined per quarter of year) as an offset term [90] into the model. Furthermore, we controlled for repeated sampling of the same individuals, communities and quarters by including these factors as random intercepts. In Model 1a and 1b, we nested quarter within community since there were several data points per quarter per community. In addition, in Model 1b quarter was nested within subject because there might be several data points per subject during the same quarter. Finally, we included random slopes for sex within community and sex within quarters to account for the possibility that sex preferences might vary across time (i.e. across quarters) within a community and between study communities within a species (table 2) and to keep the type I error rate at the nominal level of 0.05 [91,92].

### 2.4. Species differences in the determinants of top and significant associates

To explore whether the sex of the top and significant associates reflected sex-specific preferences of males and females of each species or whether these preferred associations were rather a by-product of preferences for associating with maternal kin, we fitted a second set of models. For all possible dyads of all individuals present in each community during a given quarter (or year for Lomako), we assessed whether individual 2 in the dyad was the top and/or a significant associate of individual 1. We then fitted two logistic GLMMs to a dataset comprising all possible dyads within each quarter, one with ‘Was individual 2 the top associate of individual 1 during a particular quarter (Y/N)’ as response variable (Model 2a: what-makes-top-associates model) and one with ‘Was individual 2 a significant associate of individual 1 during a particular quarter (Y/N)’ as response variable (Model 2b: what-makes-significant-associates model). As test predictor variables we included: (i) species, (ii) the three-way interaction between sex of individual 1, sex of individual 2 and species to test whether chimpanzee and bonobo males and females differed in the sex of their top and significant partners, (iii) the two-way interaction between maternal kinship and species to test for the effect of kinship on association patterns and to assess whether this effect is more important in one species than in the other and (iv) the two-way interaction between species and being a top (Model 2a) or significant (Model 2b) associate during the previous quarter (or year in the case of Lomako), since the effect of stable dyadic association patterns shown in both species [21,71] could affect the observed association pattern differently in each species.

In both models, we controlled for repeated sampling over the same individuals, communities and dyads by including them as random intercepts. In addition, we included a series of random slopes (details in table 2) to account for the possibility that the effects of sex, kinship and previous top/significant associate varied across individuals within the same community and across communities within the same species.

### 2.5. Species difference in the differentiation of pairwise affinity value within each sex combination

After characterizing sex-specific association patterns in general in chimpanzees and bonobos, we aimed to assess the degree of differentiation in association with different partners for each individual towards individuals of the same sex and of the opposite sex, respectively. We quantified the degree of differentiation of the PAVs for each individual within each sex combination using a skew index. This index was calculated as follows [93]:

$$SK_{\mathrm{PAV}}=\frac{\mathrm{sum}{\left(\mathrm{PAV}-\mathrm{mean}\;\mathrm{PAV}\right)}^{3}}{n-1}.$$

The skew index reflects the overall shape of the distribution of the PAVs and therefore relates to how differentiated the association of a given individual towards all other individuals of a given sex were. A high SK_PAV_ indicates that an individual associated a lot with few specific individuals and little with all others, and a low SK_PAV_ indicates that an individual did not associate particularly strongly with particular individuals. To test for species differences in the differentiation of PAVs according to the sex combination of dyads, we fitted an LMM (i.e. Gaussian error structure and identity link function) with the SK_PAV_ for each individual with a given sex as response variables (Model 3). In Model 3, we included as a test predictor the three-way interaction between the sexes of both partners in a dyad and species. Individual, community and quarter (nested within community and nested within individual) were included as random intercepts. We included a series of random slopes (detailed in table 2) to account for the possibility that the effect of sex combination on SK_PA_ values varied across individuals within the same community and across communities within the same species.

### 2.6. General considerations

All models were fitted three times each, each once with the PAVs, top and significant associates derived from the three different randomization methods (see the electronic supplementary material). The type of randomization had no impact on the general conclusions drawn from the various models (electronic supplementary material, tables S2–S7), and we therefore report only the results from the models fitted on the top and significant associates and the PAVs derived from the ‘individual randomization’ in the result section. To avoid cryptic multiple testing [94], we compared each full model with a respective null model lacking species and the interactions it was involved in but being otherwise identical to the full model.

All models were fitted in R ([95], v. 3.3.1) using the function ‘lmer’ of the R-package lme4 ([96], v. 1.1-12). Sample sizes are reported in the tables associated with the results section. We fitted Model 3 using maximum likelihood (lmer argument REML set to FALSE to enable likelihood ratio tests). All full model comparisons as well as all tests of individual predictors were based on likelihood ratio tests [91,97]. For Gaussian models (Model 3), we checked whether the assumptions of normally distributed and homogeneous residuals were fulfilled by visual inspection of a QQ-plot [98] and residuals plotted against fitted values [99], which revealed no heavy violations of these assumptions.

## 3. Results

### 3.1. Species differences in the sex of the top and significant associates

**Figure 1.**
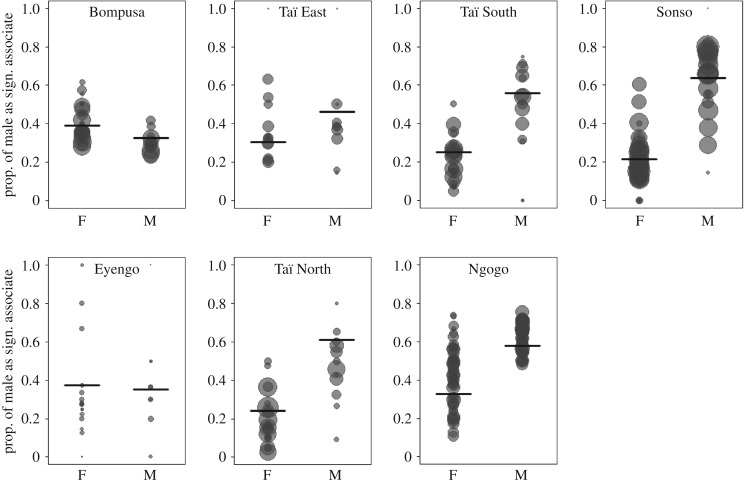
Proportion of males as significant associates averaged for each individual over all the three-month periods for males (M) and females (F) in each study community. Each dot represents an individual and the area of the dot is proportional to the number of three-month periods during which a given individual was observed. The darker the dots, the more data points overlay on this value. The horizontal segments indicate the fitted value resulting from Model 1b.

Our model investigating the sex of the top (Model 1a) and significant (Model 1b) associates were both significantly different from the respective null models (full–null model comparisons in table 3). We found a clear species difference between chimpanzees and bonobos in the sex of the top and significant associates for individuals of a given sex (tests of the interaction sex × species in table 3). While in chimpanzees, most top and significant associates were of the same sex, in bonobos females did not have a strong tendency towards having more females than males as top and significant associates and males had mostly females as top and significant associates (figure 1; electronic supplementary material, figure S2).

**Table 3.** Results of the ‘sex-top-associate’ (Model 1a) and ‘sex-significant-associates’ (Model 1b) models fitted to test for species differences in the sex combination of top and significant associates. Significant *p*-values are indicated in italics. *p*-Values are only given for terms not included in an interaction. Results are based on PAV values derived from the ‘individual randomization’. Results for other randomizations are provided in electronic supplementary material, tables S2 and S5.

	Model 1a sex-top-associate			Model 1b sex-significant-associates		
sample size	number of top associates across all quarters = 3892, number of unique individual ID = 314, number of quarter ID = 200, number of communities = 7			number of significant associates across all quarters = 17 545, number of unique ID = 312, number of quarter ID = 312, number of communities = 7		
null versus full model	*χ*^2^	d.f.	*p-*value	*χ*^2^	d.f.	*p-*value
	22.335	2	*<0.001*	10.779	2	*0.005*
	estimate ± s.e.	*χ*^2^	*p-*value	estimate ± s.e.	*χ*^2^	*p-*value
intercept	0.60 ± 0.27			−0.50 ± 0.19		
sex (male)	−0.64 ± 0.47			−0.20 ± 0.36		
species (chimpanzee)	−1.50 ± 0.30			−0.54 ± 0.22		
sex × species	3.21 ± 0.53	15.41	*<0.001*	1.52 ± 0.41	7.62	*0.006*

### 3.2. Species differences in the determinants of top and significant associates

In our second set of models, we tested whether the species differences in sex-specific association patterns still hold when maternal kinship and stability of association partners are controlled for while analysing what parameters are associated with significant associations among dyads in a given quarter. For both models, the full–null model comparison was clearly significant (table 4).

**Table 4.** Results of the ‘what-makes-top-associates’ (Model 2a) and ‘what-makes-significant-associates’ (Model 2b) models fitted to test for species differences in the characteristics of top and significant associates for individuals of each sex. Significant *p*-values are indicated in italics. *p*-Values are only given for terms not included in an interaction. Results are based on PAV values derived from the ‘individual randomization’. Results for other randomizations are provided in electronic supplementary material, tables S3 and S6.

	Model 2a what-makes-top-associate			Model 2b what-makes-significant-associates		
sample size	total number of periods (quarter or year) = 47 845, number of dyads = 7055, number of individuals = 302, number of communities = 7.					
null versus full model	*χ*^2^	d.f.	*p-*value	*χ*^2^	d.f.	*p-*value
	33.81	6	*<0.001*	43.33	6	*<0.001*
	estimate ± s.e.	*χ*^2^	*p-*value	estimate ± s.e.	*χ*^2^	*p-*value
intercept	−3.82 ± 0.42			−2.34 ± 0.39		
Sex 1 (male)	−0.55 ± 0.28			−0.04 ± 0.15		
Sex 2 (male)	−0.22 ± 0.32			−0.06 ± 0.15		
species (chimpanzee)	0.05 ± 0.49			0.54 ± 0.46		
kin (Yes)	3.67 ± 0.27	21.28	*<0.001*	3.83 ± 0.58		
top/significant associates in previous quarter (Yes)	1.62 ± 0.12	20.99	*<0.001*	0.74 ± 0.09	15.85	*<0.001*
kin × species				−1.37 ± 0.68	3.01	0.083
Sex 1 × Sex 2	−0.23 ± 0.48			−0.17 ± 0.27		
Sex 1 × species	−0.42 ± 0.31			−0.52 ± 0.17		
Sex 2 × species	−0.68 ± 0.37			−0.50 ± 0.17		
Sex 1 × Sex 2 × species	2.72 ± 0.52	27.83	*<0.001*	1.84 ± 0.29	38.31	*<0.001*

In the ‘what-makes-top-associate model’ (Model 2a), the interactions between ‘was the top associate in the previous quarter (Y/N)’ and species (likelihood ratio test: *χ*^2^ = 1.33, d.f. = 1, *p* = 0.53) and between maternal kinship and species (*χ*^2^ = 1.59, d.f. = 1, *p* = 0.33) were both not significant, indicating no significant species difference in the stability of associations and in the influence of close maternal kinship on top associates.

In the ‘what-makes-significant-associates model’ (Model 2b), the interactions between ‘was a significant associate in the previous quarter (Y/N)’ and species was not significant (average *χ*^2^ = 0.40, d.f. = 1, average *p* = 0.53) but the interaction between maternal kinship and species approached significance (*χ*^2^ = 3.08, d.f. = 1, *p* = 0.079). We refitted Model 2a and 2b without the clearly non-significant interactions (*p* > 0.3) but keeping the interaction between species and kinship in Model 2b.

In both species, maternal kin were more likely to be top associates than other individuals (average *χ*^2^ = 21.28, d.f. = 1, average *p* < 0.001, Model 2a, table 4). Maternal kinship also favoured significant associates in both species but the effect of maternal kinship on significant association tended to be stronger in bonobos than in chimpanzees (interaction kinship × species in Model 2b, average *χ*^2^ = 3.01, d.f. = 1, average *p* = 0.083; table 4; electronic supplementary material, figure S3). In both species, being top and/or significant associate of a given individual during a three-month period (or during a given year for Lomako bonobos) increased the likelihood of being a top and/or significant associate in the following three-month period (Model 2a: average χ^2^ = 20.99, d.f. = 1, average *p* < 0.001; Model 2b: average χ^2^ = 15.83, d.f. = 1, average *p* < 0.001; table 4).

**Figure 2.**
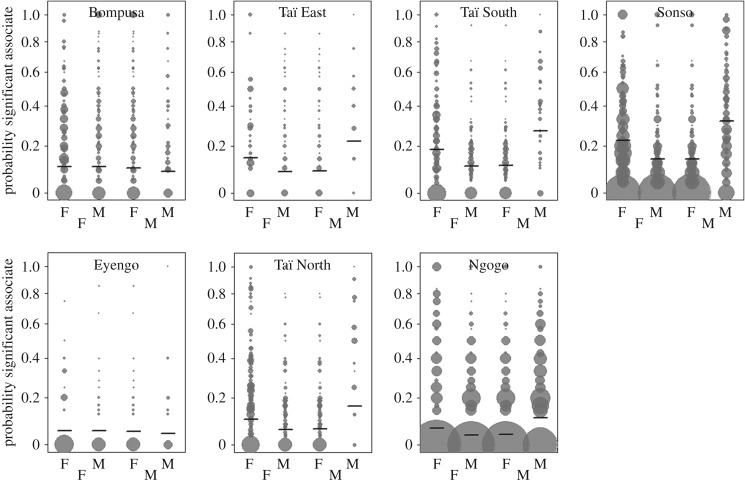
Proportion of three-month periods that a given dyad was significantly associated, separately for each sex combination (for each graph from left to right: females towards females, females towards males, males towards females and males towards males). Each dot represents a dyad and the area of the dot is proportional to the number of three-month periods during which a given dyad was observed. The horizontal segments indicate the fitted value resulting from Model 2b (controlling for maternal kinship and stability of association).

Finally, in line with the results from the ‘sex-of-top/significant-associates models’, we found that the three-way interaction between sex of individual 1, sex of individual 2 and species was significant in Model 2a and 2b (Model 2a: average χ^2^ = 27.83, d.f. = 1, average *p* < 0.001; Model 2b: average χ^2^ = 38.42, d.f. = 1, average *p* < 0.001, table 4), indicating that the sex combination of dyads most likely to be the top and significant associates differed between the species. In fact, when controlling for the effects of close maternal kinship and stability of top/significant associates, bonobos did not exhibit a strong preference for one sex over the other (figure 2; electronic supplementary material, figure S4). By contrast, in chimpanzees, males were more likely to have males than females as top (electronic supplementary material, figure S4) and significant (figure 2) associates, and females were more likely to have females than males as significant associates (figure 2).

### 3.3. Species difference in the differentiation of pairwise affinity value within each sex combination

Model 3, testing for species differences in the sex-specific skew of associations, was significantly different from the corresponding null model (table 5). We found a significant species difference in how differentiated the associations within each sex combination were (tests of the interaction sex individual 1 × sex individual 2 × species in table 5).

**Table 5.** Results of the ‘association-skew’ model (Model 3) fitted to test for species differences in the differentiation of association patterns for each individual towards individuals of the same sex and of the opposite sex, respectively. Significant *p*-values are indicated in italics. *p*-Values are only given for terms not included in an interaction. Results are based on PAV values derived from the ‘individual randomization’. Results for other randomizations are provided in electronic supplementary material, tables S4 and S7.

	Model 3 association-skew		
sample sizes	number of all individuals across all quarters = 7673; number of unique individual ID = 315; number of quarter ID = 200, number of communities = 7; number of quarter ID within individual ID = 3897		
null versus full model	*χ*^2^	d.f.	*p-*value
	25.76	4	*<0.001*
	estimate ± s.e.	*χ*^2^	*p-*value
intercept	0.61 ± 0.39		
Sex 1 (male)	0.50 ± 0.25		
Sex 2 (male)	−0.05 ± 0.18		
species (chimpanzee)	0.81 ± 0.46		
Sex 1 × Sex 2	−0.67 ± 0.27		
Sex 1 × species	−1.21 ± 0.28		
Sex 2 × species	−0.90 ± 0.21		
Sex 1 × Sex 2 × species	1.35 ± 0.30	10.95	*<0.001*

**Figure 3.**
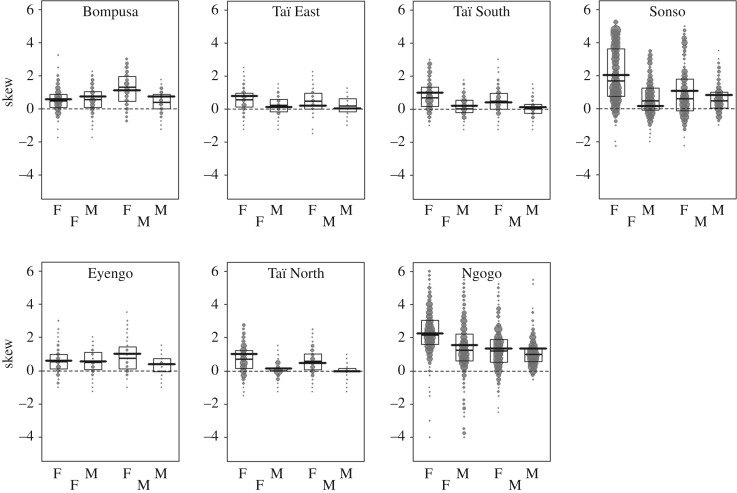
Differentiation (SK_PAv_) of association values for individuals of a given sex (M and F at the lower part of each graph) towards members of the sex indicated above (for each graph from left to right: females towards females, females towards males, males towards females and males towards males). Higher values indicate stronger skew reflecting fewer top associates. Each dot represents an individual and the area of the dot is proportional to the number of quarters (or years) it was observed. The thin black horizontal line indicates the median of the raw data and the thick black horizontal line indicates the fitted value for each sex combination based on the output of Model 3. The black box indicates the first and third quartile of the data in each sex combination for each community.

In chimpanzees, the most skewed associations were from females towards other females in all communities, indicating that females associated intensely with a few females only and little with other females (figure 3). In all chimpanzee communities, the least skewed associations were from males towards other males, indicating that males associated more uniformly with other males of the community than they did with females (figure 3).

By contrast, in both bonobo study communities, the most skewed associations were from the males towards females, indicating that males associated strongly with a few females only and little with other females (figure 3). The skews in association patterns of males towards other males and of females towards males but also towards other females were similarly low (figure 3). The latter indicates that males associated more uniformly with males than they did with females and that, compared with males, females associated more uniformly with each sex.

## 4. Discussion

Our results, based on long-term datasets on five forest-living chimpanzee and two bonobo communities, revealed a clear species difference in sex-specific association patterns between chimpanzees and bonobos (see table 6 for a summary of the results). Interestingly, the general pattern was consistent within species, despite clear differences in group size, sex ratio, demography and gregariousness across study sites ([23,100], table 1). In all five chimpanzee communities, including both eastern and western chimpanzees, association patterns were clearly sexually segregated, with both males and females having primarily same-sex partners as top and significant associates. By contrast, no such segregation was found in bonobos. In bonobos, females’ top and significant associates were more often females than males, but the difference was not as pronounced as for female chimpanzees, and male bonobos’ top and significant associates were primarily females. Interestingly, after controlling for kinship, sex-specific association patterns held in chimpanzees but not in bonobos (i.e. in bonobos neither males nor females were more likely to have top or significant associates of a particular sex). Finally, our results also highlighted species differences in the differentiation of association partners. Within species, the association patterns were the most differentiated from females towards other females in chimpanzees and from males towards females in bonobos. Overall, the strong tendency of male chimpanzees to associate with other males and the association bias of bonobo males towards females are in line with the proposed hypothesis that association preferences are driven by the individual's willingness to associate with the potentially best cooperation partners [21].

**Table 6.** Summary of the similarities and differences in parameters affecting association patterns in bonobos and chimpanzees. M: males, F: females, ‘X → Y’ indicates from X towards Y (e.g. M → F = from males towards females). TA: top associates. SA: significant associates. ∼: similar.

			species		
parameters			bonobos	chimpanzees	differences/interpretation
sex combination	not controlling for kinship	TA	M–F > M–M F–F > F–M	M–M > M–F F–F > F–M	In both species, females were more often significant and top associates of other females than males. The species, however, differed for males’ top and significant associates, being primarily males in chimpanzees and primary females in bonobos.
		SA	M–F > M–M F–F > F–M	M–M > M–F F–F > F–M	
	controlling for kinship	TA	M–M ∼ M–F ∼ F–F	M–M > F–F & F–M	When controlling for kinship, the sex-specific association pattern holds for chimpanzees, with males primarily associating with other males and females with females. However, the pattern changed for bonobos and neither male nor female bonobos were more likely to have top or significant associates of a particular sex, after controlling for kinship.
		SA	M–M ∼ M–F ∼ F–F	M–M > F–F > F–M	
association skew	most skewed associations		M → F	F → F	A high association skew indicates that individuals were highly differentiated in their association partners of a given sex, i.e. they associate strongly with a few partners and weakly with the others. Conversely, a low association skew indicates that individuals were little differentiated in their association partners of a given sex, i.e. they associated relatively equally with all individuals. The sex combination with the most skewed (i.e. differentiated) association distribution differed between the two species. In chimpanzees, the most differentiated associations were from females towards other females and the least differentiated association from males towards other males. For bonobos, associations were more differentiated from males towards females compared with all other sex combinations.
	least skewed associations		M → M & F → F	M → M	
associate in the past			positive effect on association		Individuals' association in the past had a similar effect on current association in both species, indicating that association patterns were as stable in bonobos as in chimpanzees.
kinship			positive effect on association (stronger in bonobos)		Kinship had a positive influence on association patterns in both species, but the effect tended to be stronger in bonobos, indicating that kinship might structure associations more in bonobos than in chimpanzees.

In both eastern and western chimpanzees, males engage in broad-scale cooperative actions with each other (i.e. three or more individuals cooperate to achieve a common goal) such as border patrolling [58], communal territory defence [58,101] and group hunting [102,103]. Territory defence is probably one of the most risky tasks for a chimpanzee since inter-group conflicts can be lethal [49,59]. Even though females sometimes join in inter-group conflicts [101,104,105], securing a community territory is mainly undertaken by males and being with several other males at the time of conflict may provide a clear advantage (imbalance of power hypothesis [38]). Given the risky nature of inter-group encounters in chimpanzees, it is, therefore, not surprising that males seek the presence of other males in their party. This need might drive the association patterns observed in our study in which males’ significant associates were consistently more likely to be males than females across the different field sites. Males also showed less differentiation in terms of which males they associate with compared with females, who were more likely to associate with specific females, a result also found previously for Ngogo [76]. Interestingly, the pattern is preserved even in Taï western chimpanzees where lethal inter-group conflicts are very rare [59]. Males’ tendency to associate primarily with other males might also be driven by the benefits derived from hunting as a group since the number of male participants in a hunt increases the likelihood of success in both eastern and western chimpanzees [23,44,103,106]. However, given that the hunting success, at least in some eastern chimpanzee populations, increases with the presence of good hunters in larger male parties, hunting might be less likely to explain such a general male affinity [107].

Finally, males can benefit from the presence of specific male partners in their party, also during within-group competition, by forming coalitions against other males. This may allow them to overpower higher ranking males, thereby gaining extra mating opportunities [108,109].

While in chimpanzees, male preference for other males as associates is clear and has been unambiguously found across study sites [21,23,62–65], past investigation of females’ tendency to associate more with males or females led to inconsistent results [7–68]. Our results showed that, across the five study communities, including both eastern and western chimpanzee populations, female chimpanzees had more females than males as significant associates, suggesting a universal tendency for female chimpanzees to selectively associate with females. This general tendency could be driven by female avoidance of frequent harassment and aggression received from males [110–114]. However, this avoidance cannot explain the fact that association patterns were the most differentiated from females towards other females (compared with all other sex combinations, table 6). This result contrasts with those published from a different chimpanzee population, Kalinzu, Uganda, where associations among females were less differentiated than those among males [65]. Gilby & Wrangham [21] argued that the apparent female chimpanzee selectivity for certain association partners over others could simply be a result of female ranging patterns. In fact, in certain chimpanzee communities each female consistently ranges within a portion (called a neighbourhood) of the community's overall home range [115], and two females in the same neighbourhood are more likely to be associating in the same party simply by chance [21]. However, this cannot explain selective female–female associations in Taï, where females have the same territorial usage as males. At least in Taï, the fact that female chimpanzees associated consistently and repeatedly over time with a subset of females might reflect female willingness to establish social bonds with specific females [116]. Such bonds may in turn promote cooperation between the females (e.g. support in conflicts) or tolerance in feeding context [116]. Finally, even in communities where females range in neighbourhoods, it has been shown that females do associate actively with specific other females beyond spatial overlap [117] and do form social bonds with each other [81,105]. Further studies, directly investigating the link between female–female association and female–female cooperation across several chimpanzee communities, are needed to determine whether different factors (i.e. overlapping female neighbourhoods in eastern chimpanzees and social bond formation in western chimpanzees) result in the same association patterns or if those patterns consistently reflect active choices of females [117].

Like females, male chimpanzees also form strong and stable social bonds with specific individuals and support each other in intra-group conflicts [36,81,118,119]. Yet males might be more labile than females in their choice of association partners and it has been suggested that they may change their association patterns tactically to either interact with others or just monitor the relationship status across other males [120]. Furthermore, alliances seem to extend beyond strongly bonded partners, and males may shift strategy across successive alliances by, for example, forming a coalition with a male they previously targeted against a male they previously supported [121,122]. Finally, the need to be with many males (see above and introduction) might overlay dyadic preferences, at least in terms of party association, resulting in the weakly differentiated dyadic association patterns from males towards other males (compared with the female–female pattern, figure 3) found in our study (and also in [76]).

For bonobos, our results also support the hypothesis that cooperation drives association patterns but, in contrast with chimpanzees, this pattern might be almost entirely driven by kinship. Our first analysis showed that male bonobos associated more than by chance with females and were more likely to have females as top associates, and females associated slightly more than by chance with other females. These results are in line with previously published bonobo research from the Lomako community [60,71]. However, after controlling for kinship, we found that sex differences largely disappeared, such that males were as likely as females to be a male's significant or top associate. The latter result is in line with another study on bonobos from Wamba which found no significant differences in the degree of association across the different sex combinations [65]. Taken together, our results indicate a strong role of mother–son preferences in structuring association patterns in bonobos which might be related to cooperation needs. In fact, those strong associations might promote coalitionary support from mothers, which leads to increased mating success of their sons [48,55–57]. Our results that, besides the strong mother–son associations, association between non-kin are sex-specific in chimpanzees but not in bonobos, might be related to species differences in the heterosexual dominance structure, the degree of group-level cooperation and the form of female–female feeding competition. In bonobos, females often occupy the highest dominance rank positions but some males outrank several females [53]. In such a society where one sex is not clearly dominant over the other, individual decisions to associate with one sex more than the other might be less crucial than as in male-dominated chimpanzee societies [49]. This seems contradictory to the general coalitionary pattern found in bonobos where most coalitions are formed between females with males as the primary targets [53,54]. Bonobo females appear to form differentiated social relationships by, for instance, selectively and consistently grooming certain females more than others [85]. However, the degree of affiliation in female–female dyads is not related to coalitionary support, and coalitions are rather opportunistic and formed with a broad range of females [53,54,85]. This loose female–female coalition pattern in bonobos might also explain the low degree of differentiation in bonobo dyadic female–female associations in our study (figure 3, table 6). The most differentiated associations in bonobos were from males to females and probably reflect associations between sons and their mothers. Another key difference with chimpanzees is that bonobos have never been reported to engage in border patrols or group hunting, and, most critically, lethal inter-group encounters were never observed [59] and inter-group encounters include cross-group same-sex affiliation [48,60]. Without clear benefits from group-level coordination during competitive or predatory contexts, unlike male chimpanzees, there is much less incentive for bonobo males or females to associate with same-sex individuals. Finally, the high abundance of non-monopolizable food, such as terrestrial herbaceous vegetation, in some bonobo compared with some chimpanzee habitats [123] might lower the degree of contest competition. As such, bonobo females may have less to gain from associating preferentially and cooperating with specific females during feeding competition than female chimpanzees.

One potential limitation of our study is that we did not control for the presence of potentially fertile females in our community, which might affect party size and the percentage and/or number of males in a party in both chimpanzees and bonobos [60,79,120,124,125]. However, most top and significant associates of male bonobos and chimpanzees were not potential sexual partners (they were other males for male chimpanzees and female kin for male bonobos), a pattern which cannot be driven by the presence or absence of potentially fertile females.

Another limitation of our study is that we did not include ecological variables in our analysis. To reduce the potential influence of ecological variation, we limited our analysis to forest-dwelling populations, but the study populations differ in some other ecological variables such as predation pressure (e.g. leopards, the main predators of chimpanzees and bonobos, are present in both bonobo field sites [126] (B.F. and G.H. 2002, personal observation) and in Taï [127], but not in Budongo and Kibale [128]). However, the significant effect of species on sex-specific association patterns was maintained even after controlling for the presence of leopards for each study site (see electronic supplementary material, tables S8 and S9). This complementary analysis revealed a significant effect of the ‘predation’ factor on the degree of sex differences in association patterns in some models. This effect could be related to the presence of leopards or to other variables which covary with leopard presence and distinguish Taï communities from the two eastern chimpanzee communities (Kibale and Budongo) such as the risk of being killed in inter- and intra-group conflicts, which is higher in eastern chimpanzees (reviewed in [59]). The parameters influencing within-species variation in association patterns should be investigated further using an extended range of chimpanzee populations living under contrasting ecological conditions (e.g. savannah and forest-living chimpanzees).

Finally, given limitations in the assessment of maternal kinship in this study, our results concerning the influence of close kinship on association patterns should be viewed with caution. We know that female chimpanzees remaining in their natal community can form strong associations with their mothers [129]. Although we expect the number of undetected mother–daughter dyads to be small, the number of those female dyads in communities potentially affects the strength of the influence of maternal kinship on association patterns.

Similar to chimpanzees and bonobos, the benefits of cooperation may drive association patterns in humans and other mammalian species living in societies with a high degree of fission–fusion dynamics. For example, in spider monkeys, males engage in communal territory defence similar to that observed in chimpanzees. The males travel along the border of their territories [42,130] and engage in raids towards the neighbouring communities [41]. In this species, there is also a tendency for males and females to primarily associate with individuals of the same sex [43,131]. This pattern seems to be driven by an active preference of males to associate with other males since male–male dyads had the highest average association indices compared with other sex combinations [132], and males associate more with each other than expected by chance [133]. The need for males to cooperate with each other against outsiders also seems to drive the association patterns in dolphins, a species in which sex segregation is pushed to its extreme since adult males and females appear to associate only for reproductive purposes [33]. Dyadic male–male associations are extremely strong within what is called a first-order alliance [134,135]. However, to successfully steal females from other male–male alliances, associated males typically need to cooperate with up to ten males within a second-order alliance [134]. Within a second-order alliance, males are much less selective in regard to their coalition partners [134] and, like male chimpanzees, appear to favour a large number of associates (potential cooperators) over strong selectivity towards a few associates. The same pattern is observed in humans in which men maintain a broader social network with same-sex peers and are less selective in their same-sex friends than women [136,137]. This has been hypothesized to be a social adaptation to the high degree of between-group conflicts observed in humans, which mostly involve men [138,139].

## 5. Conclusion

Association patterns have been extensively studied in bonobos and chimpanzees, but our study is the first to compile long-term datasets from several populations of each species. Our results are consistent across populations of the same species and corroborate the notion that species and sex differences in associations are influenced by the extent to which cooperation occurs in both bonobos and chimpanzees, respectively. In humans and other animals, theories have linked sex differences in sociality to the form and the degree of between-group conflicts [37,138]. We could show that, as predicted, chimpanzees, with intense between-group competition principally involving group-level male–male coordination, have strong but undifferentiated male–male association patterns, which are absent in bonobos with less between-group competition and a lack of group-level male–male coordination. Notwithstanding the limited comparative dimension of our study, with only two species, our results support these theories by showing behavioural flexibility in association pattern in two closely related species at the opposite end of the spectrum of between-group competition intensity.

## Supplementary Material

Supplement methods

## Data accessibility

Our data are deposited at Dryad: http://dx.doi.org/10.5061/dryad.dg154 [140].
