# Supplementary material for: Sex-specific association patterns in bonobos and chimpanzees reflect species differences in cooperation
Source: R Soc Open Sci. 2017 May 3;4(5):161081. doi: 10.1098/rsos.161081 (PMC5451801; doi:10.1098/rsos.161081)
Supplement: Supplement methods [file rsos161081supp1.pdf]

Supplement Methods:

Definitions of party composition:

While a party was defined at all sites as individuals ranging within visual contact, the recording methods differed slightly between them. In Budongo and Kibale party composition data were based on hourly scan sampling. In LuiKotale, party composition was recorded in a cumulative way (i.e., all individuals seen within a given hour in a party were included within the hourly party composition) due to the low visibility of bonobos on the ground (Hohmann and Surbeck personal observation). In Taï, party compositions were recorded continuously and hourly party compositions were extracted from the original data following methods used in LuiKotale. While these different methods might cause differences in overall party sizes between the sites, they are unlikely to bias the results concerning dyadic association preferences because they do not alter individual attendance rates in parties different for different community members. Party compositions were recorded during individual focal follows at Taï and during party follows at all other field sites. Measurements on simulated data indicated that the type of follow (party follow versus individual focal follow) did not influence our measurement of dyadic association preferences (see next section).

Similarity of association data obtained from focal follow versus party follow

To test whether the different types of follows (party follow or focal follow) could influence our measurement of dyadic association preferences, we simulated parties comprising 1 to 16 subjects (with all subjects being included with equal probabilities), with 10 subjects considered 'focals' (which could be the focal individual during the party composition assessments) and 6 being 'non-focals' (which never were considered focal individuals during the party composition assessments) whereby each party comprised at least one focal. We then defined six pairs of subjects to be preferred associates (two comprising only focals, two comprising only non-focals and two comprising one focal and one non-

focal). We assigned a focal to each party, randomly chosen out of the potential focals in the party. After generating party compositions, we simulated preferred associations as follows:

For each dyad of preferred partners, we selected the parties in which one was present and the other not. We then randomly selected half of these parties, removed one of the individuals present in it which was not member of the dyad and replaced it by the missing partner. We simulated 300 to 1000 parties (with an increment of 100). For each of the parties we estimated dyadic association (simple ratio index), once considering all dyads present in the party, once considering only dyads involving the respective focal, and once considering all dyads except those involving the focal. Note that the second associations can, by definition, not include dyads consisting of non-focals only. We then determined for each individual its most preferred partner and compared this with the simulated preferred partner. We found that the three methods of data collection produced largely corresponding results in that the preferred partners were identified almost always correctly when using 800 party compositions (see figure S1). Using all the party attendances from an individual (independent of it being the focal individual of observation) allows with a smaller number of party compositions to correctly infer about the preferred partner within a social group. Consequently, we felt legitimated to use all the data on party compositions to infer about dyadic preference of all individuals in the community and not only use those from the focal individual at the time of party composition assessment to infer about the focal’s preferences.

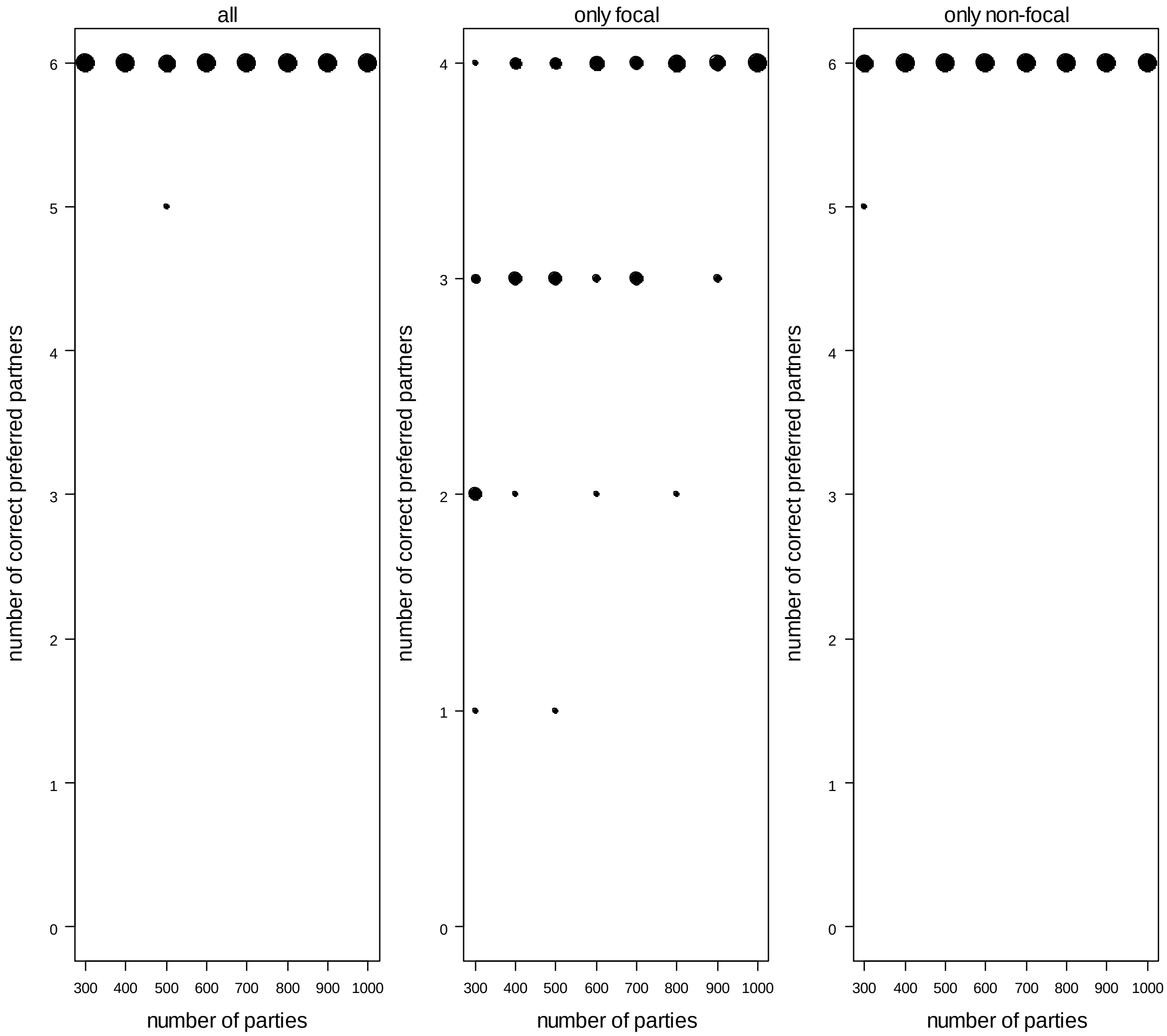

Figure S1: Results of the simulations conducted to estimate the number of preferred partners correctly assigned based on association indices derived using all dyads present in a given party, derived using only dyads involving the respective focal, and using only dyads not including the respective focal.

The three randomization methods used to extract the PAV values

The first randomization algorithm ('individual randomization') kept the observed party sizes and the number of observations per individual constant. Furthermore, by randomizing individuals only within

parties of the same size, this algorithm also kept the individual gregariousness constant. Keeping the number of observations per individual constant, we accounted for individual differences in observation frequencies. While a smaller number of observations reduces the power to detect a significant association, it does not influence the measures we used (top associate, sex ratio among significant associates).

By shuffling blocks of continuous party presence/absence of individuals, a second randomization algorithm ('block randomization') kept constant the autocorrelation in the data due to the presence of individuals in consecutive party scans. While this algorithm kept the individual number of observations constant, it neither accounted for individual differences in gregariousness nor for community specific party sizes.

In a third approach ('subset randomization'), we used the first randomization algorithm but in addition also accounted for the autocorrelation between consecutive party scans. This was done by analyzing only a subset of the original data. The subset was randomly drawn from the original data with a mean sample interval that corresponded to the mean number of consecutive scans that two individuals were observed together in the same party in a given population.

Comparing the outcome of the 3 randomizations we found a strong correlation between the PAVs of the individual randomization and the subset randomization (Spearman correlation between PAVs for each community within each quarter: mean rho = 0.93, mean $p < 0.01$). The PAVs derived from the "block randomization" were less correlated with the ones derived from the other two randomization methods (Spearman correlation between PAVs for each community within each quarter: individual randomization and "block randomization, mean rho = 0.59, mean $p < 0.01$; subset randomization and block randomization mean rho = 0.56, mean $p < 0.01$). When comparing the number of significant associates, we found that the subset randomization method was the most conservative (i.e., the randomization

method which gave the lowest number of significant associates per individual within each community within each quarter) followed by the “individual randomization” and the “block randomization” (see Table S1). For our conclusions on species differences in association patterns we considered the outcomes of all randomization procedures which produced largely consistent results (but present only the outcomes of the individual randomization in the main manuscript).

Table S1: Average number of significant associates per individual in each community within each quarter for each randomization procedure.

	Average number of significant associates per individual		
**Community**	**Individual randomization**	**Blockwise randomization**	**Subset randomization**
Taï North	2.1	4.7	0.2
Taï South	2.8	8.0	0.1
Taï East	3.9	6.7	0.2
Sonso	5.6	18.4	1.8
Ngogo	9.0	19.5	1.0
Eyengo	1.2	2.4	0.5
Bompusa	4.8	5.5	1.9

Removing double entries in Model 2a and 2b (characteristics of the top/significant associates)

Due to the nature of the association index, the data used for models 2b comprised a considerable proportion of significant associations twice (since each dyad was included twice in the data, with individual 1 and 2 in both orders). Since keeping these double entries in the data would artificially inflate the sample size, we randomly selected only one value per dyad and quarter. In order to avoid any bias due to any particular random selection we repeated the procedure 100 times and report the average results. Although being top associate is not necessarily symmetric in the sense that when A is top

associate of B, B is also the top associate of A, we applied the same random selection as for model 2b also for model 2a to avoid the necessity of including a random effect for the combination of quarter and dyad which would likely have led to convergence problems since in the far majority (97%) of combinations of dyad and quarter the response was invariably both 0 (neither of the two is top associate of the other) or both 1 (each of the two is top associate of the other).

Data transformation in Model 3 (skew of the PAV values) to fit Gaussian model assumptions

Since the residuals of Model 3 severely deviated from the assumptions of being normally distributed and homogeneous and since transformations of the response could not alleviate this issue (because of both tails of the response being 'heavy'), we transformed the skew index as follows: We replaced each skew value with its respective quantile, assuming a normal distribution with a mean of 0 and a standard deviation of 1 and a size equaling the number of skew value's (Legendre & Legendre, 1998).

Tests of the assumption for all the statistical models

For models 1 and 3 we tested for model stability by excluding levels of the random effects one at a time and this revealed robust results. For model 2 we did not estimate model stability because this was computationally not feasible. However, since the sample size was large and the effects found were strong it seems unlikely that this model appears unstable. Collinearity between the different predictor variables was not an issue since all the Variance Inflation Factor values (VIF; Field 2005) were below 1.1 for all models.

Table S2: Results of the "sex-top-associates" (Model 1a) and "sex-significant-associates" (Model 1b) models fitted to test for species differences in the sex of top and significant associates. Significant p-values are indicated in bold. P-values are only given for terms not included in an interaction. Results are based on PAV values derived from the "**block randomization**".

	**Model 1a**<br>**(block randomization)**<br>Sex of the top associate			**Model 1b**<br>**(block randomization)**<br>Sex of the significant associates		
**Sample size**	number of top associates across all quarter = 3932, number of unique individual ID = 314, number of quarter ID = 203, number of communities = 7			number of significant associates across all quarter = 42348, number of unique individual ID = 314, number of quarter ID = 3672, number of communities = 7		
**Null vs. full model**	χ^2	*df*	*P*	χ^2	*Df*	*P*
	21.02	2	**<0.001**	6.02	2	**0.049**
	Estimate±SE	χ^2	*P*	*Estimate±SE*	χ^2	*P*
Intercept	0.50±0.25			-0.49±0.21		
Sex (male)	-0.64±0.55			-0.05±0.16		
Species (chimpanzee)	-1.20±0.28			-0.04±0.24		
Sex*Species	3.21±0.64	11.37	**<0.001**	0.55±0.18	6.01	**0.014**

Table S3: Results of the "what-makes-top-associate" (Model 2a) and "what-makes-significant-associates" (Model 2b) models fitted to test for species differences in the characteristics of top and significant associates for individuals of each sex. Significant p-values are indicated in bold. P-values are only given for terms not included in an interaction. Results are based on PAV values derived from the "**block randomization**".

	**Model 2a** (block randomization) Characteristics of the top associate			**Model 2b** (block randomization) Characteristics of the significant associates		
**Sample size**	total number of periods (quarter or year)= 47845, number dyads = 7055, number individuals = 302, number of communities = 7.					
**Null vs. full model**	χ^2	*df*	*P*	χ^2	*df*	*P*
	33.81	6	**<0.001**	26.58	6	**<0.001**
	Estimate±SE	χ^2	*P*	*Estimate±SE*	χ^2	*P*
Intercept	-3.77±0.43			-1.91±0.53		
Sex 1 (male)	-0.50±0.30			0.06±0.28		
Sex 2 (male)	-0.20±0.24			0.05±0.28		
Species (chimpanzee)	0.00±0.50			1.07±0.63		
Kin (Yes)	3.58±0.31	19.42	**<0.001**	3.24±0.55		
Top/significant associates in previous quarter (Yes)	1.43±0.12	22.47	**<0.001**	0.67±0.11	13.83	**<0.001**
Kin* Species				-1.43±0.65	3.05	0.083
Sex 1 * Sex 2	-0.30±0.48			0.03±0.27		
Sex 1 * Species	-0.67±0.33			-0.10±0.32		
Sex 2 * Species	-0.59±0.27			-0.09±0.33		
Sex 1 * Sex 2 * Species	2.86±0.52	30.03	**<0.001**	1.36±0.30	19.72	**<0.001**

Table S4: Results of the "association-skew" model (Model 3b) fitted to test for species differences in the differentiation of association patterns for each individual towards individuals of the same sex and of the opposite sex, respectively. Significant p-values are indicated in bold. P-values are only given for terms not included in an interaction. Results are based on PAV values derived from the "**block randomization**".

	**Model 3b** (block randomization) Skew of PAV values		
**Sample size**	number of individuals across all quarters: 7760; number of unique individual ID = 315; number of quarter ID = 203, number of communities = 7; N of quarter ID within individual ID: 3943		
**Null vs. full model**	χ^2	*df*	*P*
	18.60	4	**<0.001**
	Estimate±SE	χ^2	*P*
Intercept	0.57±0.38		
Sex 1 (male)	0.37±0.17		
Sex 2 (male)	-0.14±0.24		
Species (chimpanzee)	0.61±0.45		
Sex1 * Sex 2	-0.51±0.22		
Sex 1 * Species	-0.90±0.19		
Sex 2 * Species	-0.62±0.28		
Sex 1 * Sex 2 * Species	0.75±0.24	7.18	**0.007**

Table S5: Results of the "sex-top-associate" (Model 1a) and "sex-significant-associates" (Model 1b) models fitted to test for species differences in the sex-combination of top and significant associates. Significant p-values are indicated in bold. P-values are only given for terms not included in an interaction. Results are based on PAV values derived from the "**subset randomization**".

	**Model 1a** (subset randomization) Sex of the top associate			**Model 1b** (subset randomization) Sex of the significant associates		
**Sample size**	number of top associates across all quarters = 3913, number of unique individual ID = 315, number of quarter ID = 202, number of communities = 7			number of significant associates across all quarters = 3528, number of unique individual ID = 254, number of quarter ID = 1653, number of communities = 7		
**Null vs. full model**	χ^2	*df*	*P*	χ^2	*Df*	*P*
	19.56	2	**<0.001**	18.91	2	**<0.001**
	Estimate±SE	χ^2	*P*	*Estimate±SE*	χ^2	*P*
Intercept	0.69±0.25			-0.47+0.22		
Sex (male)	-0.82±0.45			-0.59+0.79		
Species (chimpanzee)	-1.46±0.29			-1.56+0.26		
Sex*Species	3.11±0.51	13.86	**<0.001**	2.97+0.95	4.70	**0.030**

Table S6: Results of the "what-makes-top-associate" (Model 2a) and "what-makes-significant-associates" (Model 2b) models fitted to test for species differences in the characteristics of top and significant associates for individuals of each sex. Significant p-values are indicated in bold. P-values are only given for terms not included in an interaction. Results are based on PAV values derived from the "**subset randomization**".

	**Model 2a** (subset randomization) Characteristics of the top associate			**Model 2b** (subset randomization) Characteristics of the significant associates		
**Sample size**	total number of periods (quarter or year) = 47845, number dyads = 7055, number individuals = 302, number of communities = 7.					
**Null vs. full model**	χ^2	*df*	*P*	χ^2	*df*	*P*
	35.58	6	**<0.001**	43.25	6	**<0.001**
	Estimate±SE	χ^2	*P*	*Estimate±SE*	χ^2	*P*
Intercept	-3.78±0.43			-3.82±0.33		
Sex 1 (male)	-0.45±0.25			-0.12±0.35		
Sex 2 (male)	-0.18±0.30			-0.13±0.34		
Species (chimpanzee)	0.03±0.50			-0.83±0.38		
Kin (Yes)	3.50±0.27	20.65	**<0.001**	4.25±0.74		
Top/significant associates in previous quarter (Yes)	1.57±0.13	22.14	**<0.001**	1.53±0.39	9.33	**0.003**
Kin * Species				-0.93±0.89	1.16	0.31
Sex1 * Sex 2	-0.35±0.46			-0.22±0.45		
Sex 1 * Species	-0.47±0.28			-1.44±0.42		
Sex 2 * Species	-0.64±0.34			-1.40±0.40		
Sex 1 * Sex 2 * Species	2.71±0.49	29.25	**<0.001**	2.93±0.52	27.91	**0.02**

Table S7: Results of the "association-variance" (Model 3a) and "association-skew" (Model 3b) models fitted to test for species differences in the differentiation of association pattern for each individual towards individuals of the same sex and of the opposite sex respectively. Significant p-values are indicated in bold. P-values are only given for terms not included in an interaction. Results are based on PAV values derived from the "**subset randomization**".

	**Model 3** **(subset randomization)** Skew of PAV values		
**Sample size**	number of individuals across all quarters: 7719; number of unique individual ID = 315; number of quarter ID = 202, number of communities = 7; number of quarter ID within individual ID: 3922		
**Null vs. full model**	χ^2	*df*	*P*
	25.77	4	**<0.001**
	Estimate±SE	χ^2	*P*
Intercept	0.64±0.36		
Sex 1 (male)	0.49±0.2		
Sex 2 (male)	-0.05±0.19		
Species (chimpanzee)	0.83±0.42		
Sex1 * Sex 2	-0.68±0.27		
Sex 1 * Species	-1.25±0.22		
Sex 2 * Species	-0.89±0.21		
Sex 1 * Sex 2 * Species	1.34±0.3	10.95	**<0.001**

Table S8: Results of the "sex-top-associate with predation" (Model 1a.p) and "sex-significant-associates with predation" (Model 1b.p) models. Both models had the same structure as models 1a and 1b but incorporate an additional factor, the presence or absence of leopards. This variable was binomial with value "1" for both bonobo community and all Tai communities (where leopards are present) and value "0" for Sonso and Ngogo communities (where leopards are extinct). In this new set of models we also incorporated the two way interaction between predation and sex. Significant p-values are indicated in bold. P-values are only given for terms not included in an interaction. Results are based on PAV values derived from the "**individual randomization**".

	**Model 1a.p**<br><br>Sex of the top associate<br><br>*(with predation)*			**Model 1b.p**<br><br>Sex of the significant associates<br><br>*(with predation)*		
**Sample size**	number of top associates across all quarters = 3892, number of unique individual ID = 314, number of quarter ID = 200, number of communities = 7			number of significant associates across all quarter = 17545, number of unique individual ID = 312, number of quarter ID = 3698, number of communities = 7		
**Null vs. full model**	χ^2	*df*	*P*	χ^2	*Df*	*P*
	18.05	2	**<0.001**	8.99	2	**0.011**
	Estimate±SE	χ^2	*P*	*Estimate±SE*	χ^2	*P*
Intercept	-1.07±0.40			-0.49±0.29		
Sex (male)	0.36±0.67			0.10±0.52		
Species (chimpanzee)	-1.16±0.35			-0.54±0.24		
Predation (yes)	0.59±0.30			-0.01±0.22		
Sex*Species	2.64±0.60	12.98	**<0.001**	1.39±0.43	6.34	**0.012**
Sex*predation	-1.00±0.48	4.35	**0.037**	-0.30±0.40	0.57	0.450

Table S9: Results of the "association-skew with predation" (Model 3.p) model. This model has the same structure as models 3 but incorporates an additional factor, the presence or absence of leopards. This variable was coded as in Models 1a.p and 1b.p. In Model 3.p we also incorporated the three way interaction between predation, sex of individual 1 and sex of individual 2. Significant p-values are indicated in bold. P-values are only indicated for terms not included in an interaction. Results are based on PAV values derived from the "**individual randomization**".

	**Model 3.p**<br>Skew of PAV values<br>*(with predation)*		
**Sample size**	number of individuals across all quarters: 7673; number of unique individual ID = 315; number of quarter ID = 200, number of communities = 7		
**Null vs. full model**	χ^2	*df*	*P*
	13.67	4	**0.008**
	Estimate±SE	χ^2	*P*
Intercept	2.03±0.22		
Sex 1 (male)	-0.35±0.28		
Sex 2 (male)	-0.61±0.25		
Species (chimpanzee)	0.20±0.18		
Predation (yes)	-1.42±0.16		
Sex1 * Sex 2	0.17±0.43		
Sex 1 * Species	-0.77±0.25		
Sex 1 * Predation	0.85±0.20		
Sex 2 * Species	-0.59±0.22		
Sex 2 * Predation	0.56±0.18		
Sex 1 * Sex 2 * Species	0.83±0.39	4.61	**0.032**
Sex 1 * Sex 2 * Predation	-0.81±0.31	6.28	**0.012**

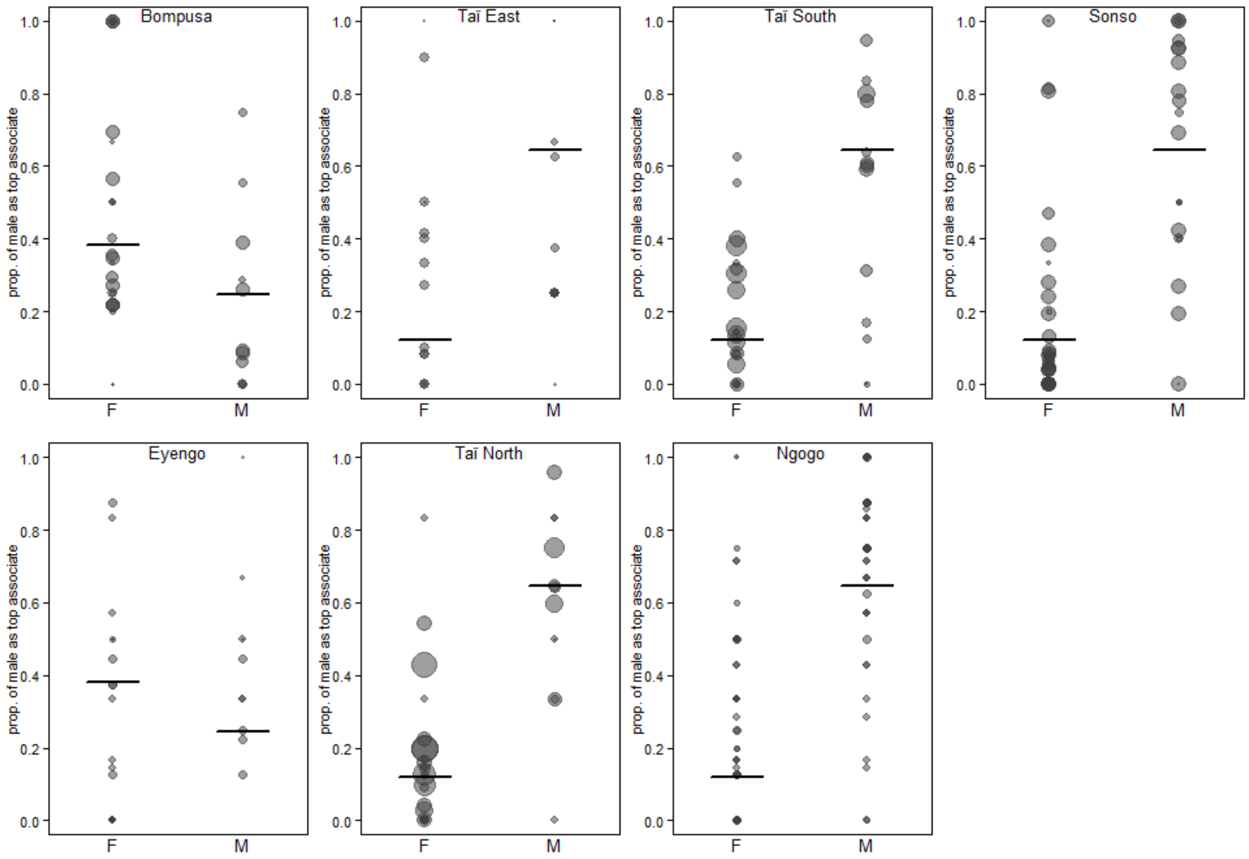

Figure S2: Proportion of three-months periods during which a male was the top associate (Y-axis) for males (M) and females (F) of each study community (based on the individual randomization)**.** Each dot represents an individual and the area of the dots is proportional to the number of three-months periods during which a given individual was observed. The darker the dots, the more data points overlay on this value. The horizontal lines indicate the fitted value resulting from Model 1a.

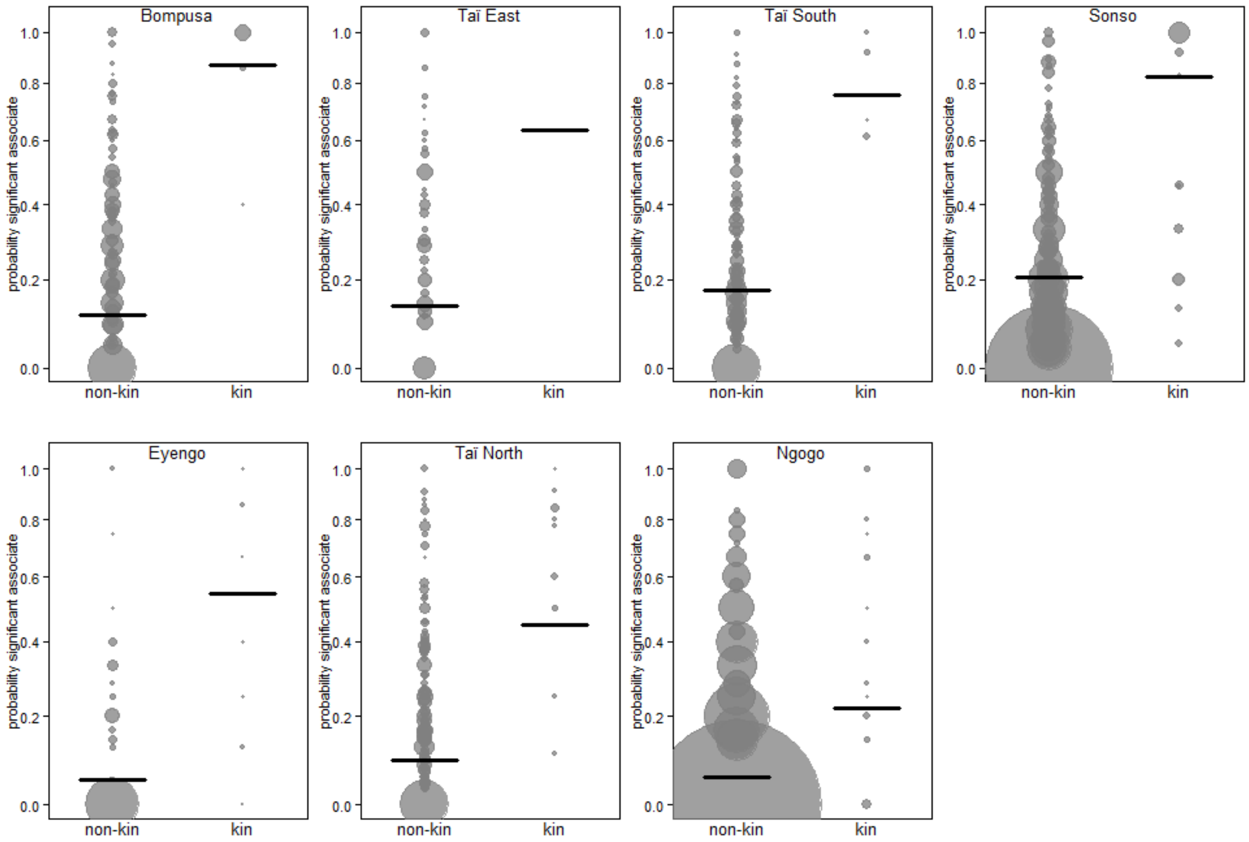

Figure S3: Effect of kinship on the proportion of three-months periods during which a given dyad significantly associated (based on individual randomization)**.** Each dot represents a dyad and the area of the dot is proportional to the number of three-months periods during which a given dyad was observed. The horizontal lines indicate the fitted value resulting from Model 2b.

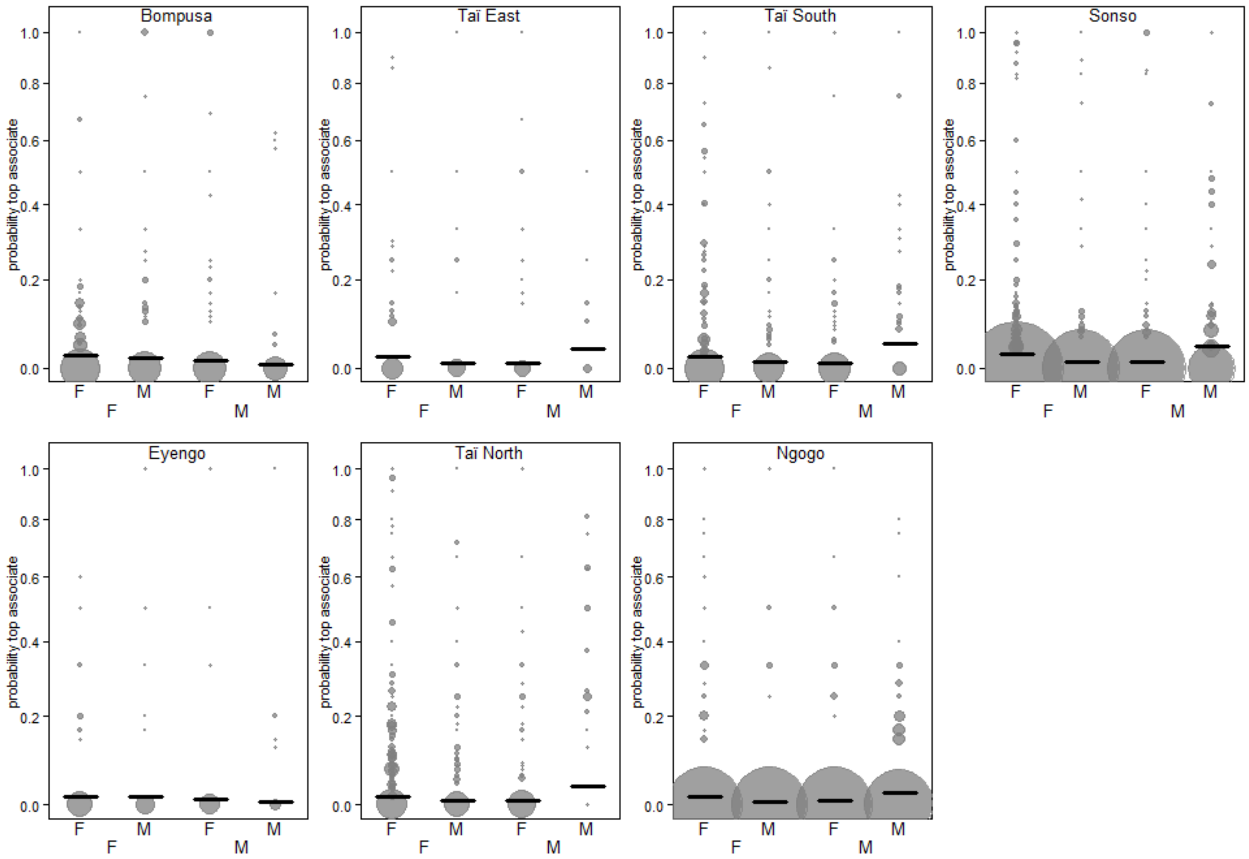

Figure S4: Proportion of 3 months periods that a given individual was top associate of another, separately for each sex-combination (based on individual randomization). Each dot represents a dyad and the area of the dot is proportional to the number of three-months periods during which a given dyad was observed. The horizontal lines indicate the fitted value resulting from Model 2a (controlling for maternal kinship and stability of association).

Supplement bibliography

Field, A. 2005. *Discovering Statistics using SPSS.* London: Sage Publications.
